# The complete mitochondrial genome of the South African snoek *Thyrsites atun* (Euphrasén, 1791) (Perciformes, Gempylidae)

**DOI:** 10.1080/23802359.2023.2179353

**Published:** 2023-02-20

**Authors:** Siphesihle Mthethwa, Aletta Bester-van der Merwe, Rouvay Roodt-Wilding

**Affiliations:** Molecular Breeding and Biodiversity Group, Department of Genetics, Stellenbosch University, Stellenbosch, South Africa

**Keywords:** Mitogenome assembly, fish, snake mackerels, phylogenetics, ion torrent

## Abstract

This is the first report on the complete mitochondrial genome (mitogenome) of South African *Thyrsites atun* (Euphrasén, 1791) and its phylogenetic placement within the Gempylidae family. The complete mitogenome of snoek is 16,494 bp in length and comprises 2 rRNAs, 13 protein-coding genes, 22 tRNAs, and one control region. Gene order is similar to that found in gempylids and other marine fishes. Reconstruction of Gempylidae phylogeny implies that the mitogenomes of snoek, black snoek *Thyrsitoides marleyi*, and snake mackerel, *Gempylus serpens* are closely related in evolutionary terms.

## 1. Introduction

**Figure 1.**
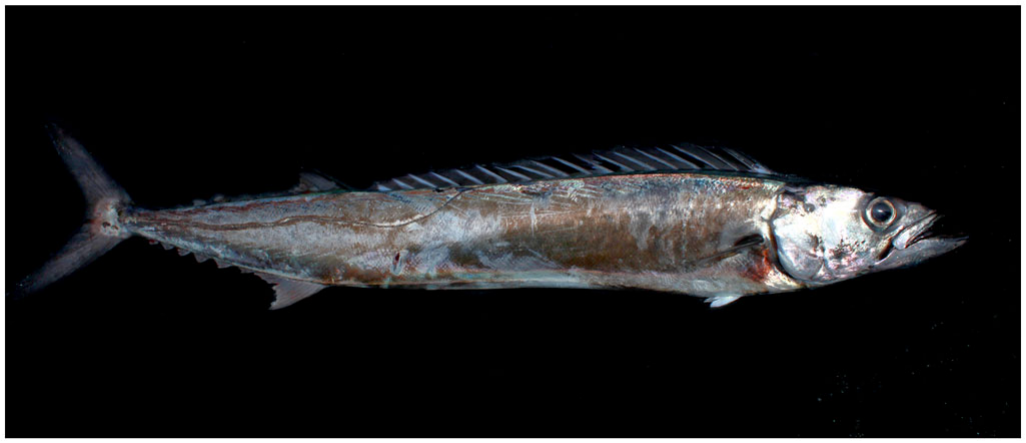
*Thyrsites atun* species reference image. Snoek has an elongated and strongly compressed body, it is dark blue with a paler belly. The key distinguishing feature of snoek is the single lateral line running close to the upper contour of the body below most of the first dorsal-fin base then abruptly curving ventrally. Photo by Brian Gratwicke distributed under license Creative Commons Attribution-Share Alike v2.0 Generic.

Fishes of the family Gempylidae are fast-swimming oceanic predators often found in the deep waters of tropical and subtropical seas (Matsubara and Iwai 1952; Nakamura 1993; Li et al. 2020; Hüne et al. 2021). The family consists of 24 species described in 16 genera (Nelson 2006; Li et al. 2020). A number of these species are economically important (Crawford and De Villiers 1985; Kailola et al. 1993; Griffiths 2002, 2003). Although the fishes of the Gempylidae have long been of interest to ichthyologists and considerable literature on this family has accumulated, the lack of consensus concerning phylogenetic relationships within this family has remained problematic (Matsubara and Iwai 1952; Johnson 1986; Carpenter et al. 1995; Li et al. 2020). In an effort to address this issue, we present here for the first time a full mitochondrial genome of the South African *T. atun* (GenBank Accession Number: OP133162). In a parallel effort, our research group assembled an additional four *T. atun* mitogenomes from the Amsterdam and Saint-Paul Islands, the Inaccessible Islands, Chile, and New Zealand, these are not described in the current paper. In teleosts, the use of mitogenomic data to resolve complex evolutionary relationships has been explicitly shown (Lavoué et al. 2005; Miya and Nishida 2015; Friedman et al. 2019). *Thyrsites atun* (Figure 1) is a medium-sized, pelagic predator distributed widely in the southern hemisphere where it supports both moderate and substantial fisheries (Griffiths 2003). In South Africa, *T. atun* (Cape snoek) is a commercially important species targeted by artisanal fisheries and commercial trawlers and constitutes a substantial portion of by-catch in teleost fisheries (Hutchings and Lamberth 2002).

## 2. Materials

The sample whose mitogenome we present was provided by the Department of Forestry, Fisheries, and the Environment (DFFE) of South Africa, Cape Town. The fin clip was collected from a snoek individual caught in St Helena Bay, South Africa (32°44’29.5” S 18°01’12.2” E) in November 2010. The fin-clip sample (stored in 99% ethanol) and DNA are kept at Stellenbosch University’s Genetics Department in South Africa under sample ID: SASN032 (http://www.sun.ac.za/english/faculty/agri/genetics, Aletta Bester-van der Merwe, aeb@sun.ac.za).

## 3. Methods

Total genomic DNA was extracted from the tissue using the cetyltrimethylammonium bromide (CTAB) extraction protocol (Sambrook and Russell 2001). Whole-genome sequencing was performed on the Ion Torrent S5^TM^ platform (ThermoFisher Scientific, Waltham, MA, USA) using 400 base-pair (bp) chemistry following the manufacturer’s protocol. An identical approach was used for all five samples. The New Zealand sample with 4,019,478 reads of 374 bp average read length post-quality filtering was the best candidate for a draft genome assembly and was thus assembled first and used as the reference genome for the subsequent samples. These reads were mapped to an incomplete mitogenome of *G. serpens* (AP012502) which is the closest relative of snoek according to a phylogeny published by Miya et al. (2013), using Geneious Prime v2020.2.5 (Biomatters Ltd., Auckland, New Zealand). The result was an incomplete draft mitogenome missing the control region. Using the SPAdes assembler v3.15.5 (Prjibelski et al. 2020) we constructed the snoek mitogenome using the *de novo* technique. When the *de novo* technique failed to construct a complete mitogenome, we took the resultant contigs and mapped them against snoek’s partial mitogenome. The contigs extended to the presumed control region resulting in a full mitochondrial genome. The newly constructed *T. atun* mitogenome, OP598802, was then utilized as a reference sequence for the remaining *T. atun* genome assemblies including the assembly of South African snoek (OP133162). Read mapping and assembly were carried out in Geneious Prime v2020.2.5 software (https://www.geneious.com) utilizing the Geneious mapper with medium/low sensitivity and fine-tuning up to five iterations, followed by manual curation.

Annotations were carried out using MitoAnnotator (Iwasaki et al. 2013) and confirmed with the MITOS Web server (Bernt et al. 2013). To investigate the placement of *T. atun* within the Gempylidae family, we performed Bayesian inference in MrBayes 3.2.7 (Ronquist et al. 2012). Thirteen protein-coding gene sequences excluding their stop codons were aligned independently and concatenated in the order in which they appear in the mitogenome producing an alignment of 11,400 bp. The nucleotide alignment was then gene-partitioned and used for tree inference. While it is common practice to select a substitution model for Bayesian phylogenetic inference using a priori model selection processes, we used the model averaging strategy instead. This strategy allows the user to choose from among all 203 alternative time-reversible rate matrices based on their posterior probability, avoiding uncertainty about the appropriate substitution model, which is an issue inherent in likelihood-based techniques (Ronquist et al. 2012; Bouckaert and Drummond 2017). The analysis was run for 10,000,000 generations with a sampling frequency of 2,000 and 25% of starting trees was discarded as burn-in. Species included in the analysis are reported in Table 1.

**Table 1.** List of mitochondrial sequences used in phylogenetic reconstruction of the family Gempylidae (Scombriformes) to investigate the placement of *Thyrsites atun* among 12 other species of gempylids. *Trichiurus lepturus* and *Benthodesmus tenuis* (Scombriformes: Trichiuridae) were used as outgroups (marked by an asterisk).

Species	Accession number	Published
*Paradiplospinus antarcticus*	AP012504	Li et al. 2020
*Diplospinus multistriatus*	AP012523	Miya et al. 2013
*Epinnula magistralis*	AP012943	Miya et al. 2013
*Gempylus serpens*	AP012502	Miya et al. 2013
*Nealotus tripes*	AP012521	Miya et al. 2013
*Lepidocybium flavobrunneum*	AP012519	Miya et al. 2013
*Nesiarchus nasutus*	AP012503	Miya et al. 2013
*Promethichthys prometheus*	AP012504	Miya et al. 2013
*Rexea nakamurai*	AP012520	Miya et al. 2013
*Ruvettus pretiosus*	AP012506	Miya et al. 2013
*Thyrsitoides marleyi*	AP012505	Miya et al. 2013
*Rexea solandri*	KJ408216	Bustamante and Ovenden, 2016
*Thyrsites atun*	OP133162	This study
Trichiurus lepturus*	MK333401	Mukundan et al. 2020
Benthodesmus tenuis*	AP012522	Miya et al. 2013

## 4. Results

**Figure 2.**
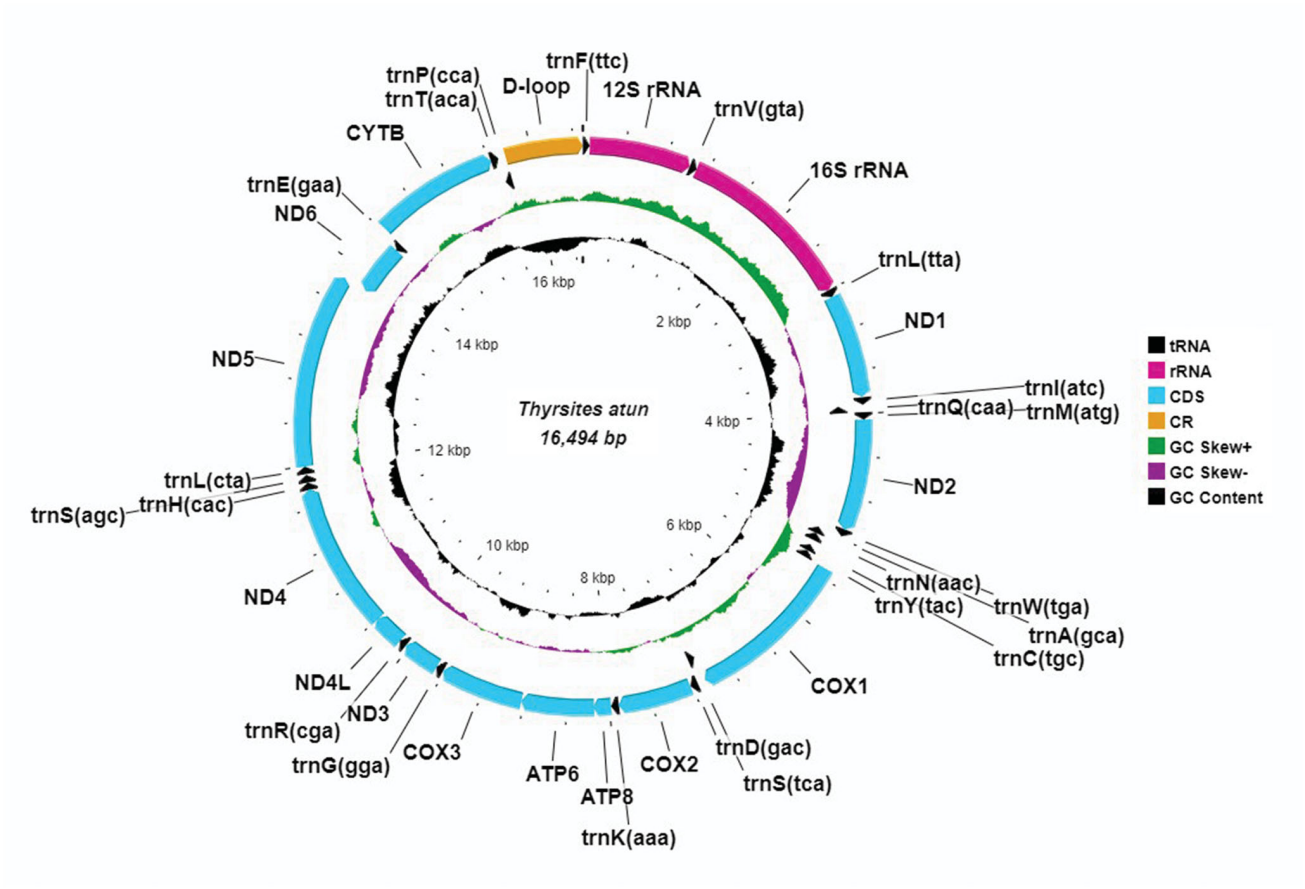
The annotated mitochondrial genome of South African *Thyrsites atun*. The complete and circular mitochondrial genome is 16,494 base pairs long, it consists of 13 protein-coding genes, 22 transfer RNA genes, 2 ribosomal RNA genes, and a control region (D-loop). Arrows indicate transcription directions.

The complete and circular mitogenome of South African *T. atun* is 16,494 bp in size and comprises 13 protein-coding regions, 22 tRNA genes, *12S*, and *16S* ribosomal RNAs, and a non-coding D-loop region. The gene arrangement and direction are typical of fishes. The *ND6* subunit gene and eight tRNAs (*trnQ(cca)*, *trnA(gca), trnN(aac)*, *trnC(tgc)*, *trnY(tac)*, *trnS(tca)*, *trnE(gaa)*, and *trnP(cca)*) are encoded on the light-strand, while the other 28 genes are encoded on the heavy-strand (Figure 2). The 13 protein-coding genes used the conventional ATN start codon except for *COX1* and *CYTB* which used GTG. The *ATP6*, *ATP8*, *COX1*, *ND1*, *ND4L,* and *ND5* genes used the TAA stop codon. The AGA stop codon was exclusive to *ND4* and ATC to *ND6*, while an incomplete stop codon TA- was found for *ND2* and *COX3*; and T– for *COX2*, *ND3*, and *CYTB*. The 13 protein-coding genes are 11,479 bp in total, and the *12S* and *16S* rRNAs are 959 and 1,701 bp, respectively. The 22 tRNA genes range from 65 bp (*trnF(ttc)*) to 75 bp (*trnK(aaa)*) in size. The mitogenome has an A + T (54.1%) bias, and nucleotide composition of A, 27.2%, T, 27%, G, 17.4%, and C, 28.5%.

## 5. Discussion and conclusion

**Figure 3.**
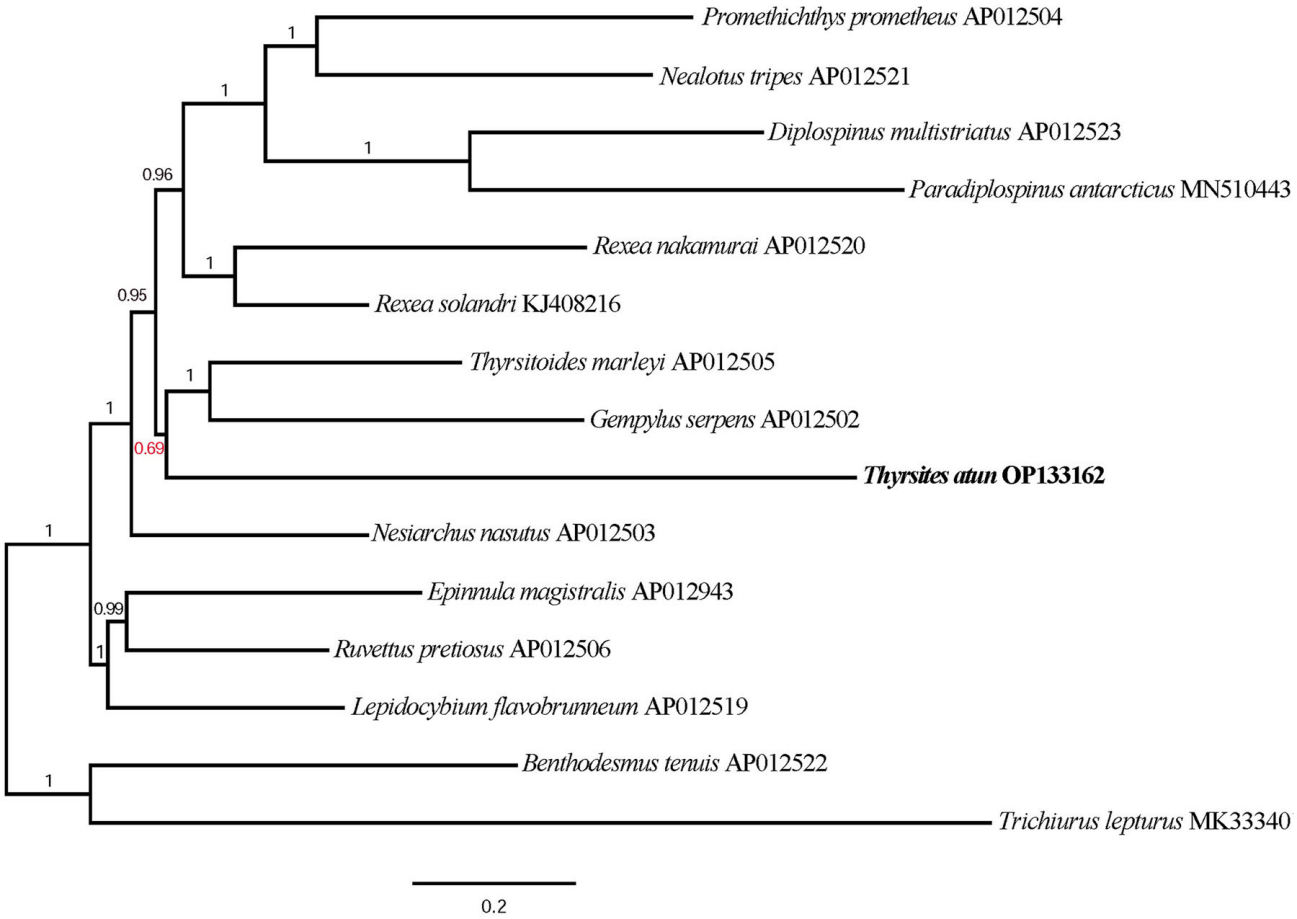
Phylogenetic tree depicting placement of *Thyrsites atun* [OP133162] within the Gempylidae family derived from a Bayesian analysis of 13 protein-coding genes. Members of Trichiuridae; *Trichiurus lepturus* [MK333401] and *Benthodesmus tenuis* [AP012522] are included as outgroups. Nodal support is indicated by Bayesian Posterior Probabilities (in red are BPP <0.95, and in black are BPP≥ 0.95).

Although this is not well supported and could be the result of unrepresentative sampling, our result suggests the mitogenome of *Thyrsites atun* is closely related to that of *T. marleyi* and *G. serpens* (Figure 3). This is in disagreement with previously published morphology-based phylogenies. Despite having a striking resemblance, *Thyrsites atun*, also known as snoek, and *Thyrsitoides marleyi*, commonly known as black snoek, are not closely related according to morphology-based phylogenies of Russo (1983) and Beckett et al. (2018). On the other hand, Gempylus and Thyrsites are the only Gempylidae species to share a carangiform swimming mode and a high number of dorsal and anal finlets, making them evolutionary close relatives (Monsch 2000).

## Data Availability

The genome sequence data that support the findings of this study are openly available in GenBank of NCBI at https://www.ncbi.nlm.nih.gov/ under accession no. OP133162. The associated BioProject, SRA, and Bio-Sample numbers are PRJNA878677, SRP396379, and SAMN30747289, respectively.
